# Deprescribing NSAIDs: The Potential Role of Community Pharmacists

**DOI:** 10.3390/pharmacy12040116

**Published:** 2024-07-24

**Authors:** Delsher Amedi, Parisa Gazerani

**Affiliations:** 1 Department of Life Sciences and Health, Faculty of Health Sciences, Oslo Metropolitan University, 0130 Oslo, Norway; 2 Department of Health Science and Technology, Faculty of Medicine, Aalborg University, 9260 Gistrup, Denmark

**Keywords:** pain, analgesics, non-steroidal anti-inflammatory drugs, NSAIDs, deprescription, deprescribing, community pharmacist, community pharmacy, pharmacist, pain

## Abstract

Non-steroidal anti-inflammatory drugs (NSAIDs) are largely used for controlling various pain conditions and are widely available in community pharmacies, with and without prescription. Despite their effectiveness, NSAIDs can pose significant risks due to potential side effects and drug interactions, particularly in polypharmacy and comorbidity contexts and for vulnerable users. This study investigated whether and how NSAIDs deprescribing can be conducted at the community pharmacy level by assessing pharmacists’ confidence, attitudes, and potential barriers and facilitators. Additionally, we aimed to identify any deprescribing guidelines that pharmacists could use. A literature search and a cross-sectional digital questionnaire targeting community pharmacists in Norway were conducted. Results showed that study participants (N = 73) feel confident in identifying needs for deprescribing NSAIDs but barriers such as time constraints, lack of financial compensation, and communication challenges were noted. Participants reported positive attitudes toward deprescribing but highlighted a need for better guidelines and training. This study highlights a gap in specific guidelines for deprescribing NSAIDs and a potential for enhancing pharmacists’ roles in the deprescribing process, for example, through training and improved financial incentives. Further research is encouraged to develop concrete strategies for an effective implementation where community pharmacists can be involved in the deprescribing of NSAIDs.

## 1. Introduction

Non-steroidal anti-inflammatory drugs (NSAIDs) such as ibuprofen, naproxen, and diclofenac play an important role in treating a wide range of pain conditions, including musculoskeletal pain [1]. Due to their widespread use, many NSAIDs are available both with and without a prescription in community pharmacies, but despite their effectiveness, NSAIDs pose significant risks [2] to patients due to possible side effects and interactions, particularly in the context of polypharmacy and comorbidity, which are popular among the elderly [3]. Deprescribing is therefore becoming a critical practice, especially in pain therapy [4]. In this context, community pharmacists can play a key role by guiding the users for rational pharmacotherapy by NSAIDs, lowering the dose, or choice of potential non-pharmacological alternatives [5]. Community pharmacists can theoretically help and as a part of the health profession design deprescribing plans in collaboration with patients and physicians [6]. Such a role has already been recognized and appreciated by hospital or clinical pharmacists and in association with many medications, including analgesics used for pain, and in particular for opioids [7]. 

Although existing literature highlights the importance of deprescription in pain management and the role of pharmacists in this process [8,9,10], there is a notable lack of research specifically focusing on the role of community pharmacists in this process [11,12]. As a result, we have formulated a research question: What opportunities and challenges exist for community pharmacists in the deprescribing of NSAIDs, with and without a need for a prescription? We aimed to explore and describe the potential role of community pharmacists in Norway in the process of deprescribing for NSAIDs in community pharmacies and to identify potential barriers and facilitators. We hypothesized that community pharmacists have the competence and potential for carrying out deprescription at the community pharmacies, and factors such as education level, work experience, and gender will not act as potential barriers. 

### 1.1. Pain and Pain Management

Pain is a complex, multidimensional phenomenon with chronic pain being a significant global health burden [13,14,15,16], affecting 19% of adults in Europe [17]. It is more common in women and older individuals and poses significant challenges, including increased mortality risk, suicide, depression, anxiety, and decreased quality of life [18,19,20,21,22,23]. Its development is influenced by genetics, environment, and various biopsychosocial factors [24]. Chronic pain can exacerbate conditions like social isolation, diabetes, cardiovascular diseases, and obesity [25,26,27]. Treatment strategies are multidisciplinary, including pharmacological and non-pharmacological methods (e.g., physical therapy, exercise, cognitive behavioral therapy, and lifestyle changes) addressing the biopsychosocial aspects of pain [28,29,30]. Non-opioid analgesics, such as paracetamol and NSAIDs, are used for mild to moderate pain but carry risks like liver damage, gastrointestinal issues, and cardiovascular risks [31,32,33].

Over-the-counter (OTC) medications like acetaminophen and ibuprofen are available in community pharmacies and some stores under European regulation, and under the LUA scheme (sales outside pharmacies scheme) in Norway [34,35,36]. Acetaminophen is a commonly used OTC and was the leading OTC medicine for fever and pain in Norway in 2022 [36]. Despite popular use, this medication carries a risk of severe liver damage at high doses or drug–drug interaction when it is one of the medications in polypharmacy conditions, especially among older adults [37,38].

### 1.2. Deprescription, Deprescribing Process, and a Potential Role of Pharmacists

Deprescribing (in Norwegian: avmedisinering [39]), the process of discontinuing inappropriate drugs to manage polypharmacy and improve patient outcomes, lacks an internationally accepted definition [4,40]. It is especially relevant for patients with comorbidities, palliative care needs, excessive polypharmacy, frailty, or declining organ function, with older patients benefiting the most [38]. Polypharmacy increases drug-related problems, leading to higher costs from unnecessary drug use and hospitalizations, and is common among older adults, younger adults with chronic conditions, and patients with mental health issues [41,42,43,44,45]. Deprescription can reduce inappropriate drug use and improve outcomes, though more evidence is needed on its clinical impact [46].

Deprescribing occurs in various settings, including hospitals and outpatient clinics, and involves regular drug reviews as utility changes over time [47]. Challenges in deprescription include managing withdrawal symptoms, finding alternative treatments, overcoming addiction, and patient resistance [48,49]. Physicians face time constraints, and patients may fear worsening conditions or withdrawal symptoms, with limited resources and competing priorities adding to the challenges in primary care [49,50,51,52,53]. Pharmacists can also play a key role in deprescription, involving medication reconciliation, patient discussions, and reviews [54,55]. They can assess the necessity of various medications including analgesics, create personalized dose reduction plans, and manage withdrawal [56]. Studies indeed support the role of pharmacists in hospital settings deprescription [56,57,58]. Pharmacist-led deprescription of oral NSAIDs in adults with chronic pain has shown improvements in function, quality of life, and pain scores [59]. 

Global deprescription initiatives involving pharmacists report significant benefits. In the Netherlands, pharmacists have achieved a successful deprescription rate of around 70% for psychotropic drugs with minimal withdrawal symptoms reported [60]. In primary care settings, pharmacist-led deprescription programs for benzodiazepines have shown promising health and economic outcomes [8,61]. In the United States, pharmacists play a crucial role in opioid deprescription through programs like STORM, leading to significant reductions in opioid use and improved patient-reported pain management outcomes [62]. In Lebanon, a study highlighted the efficacy of pharmacist-led medication review interventions aimed at deprescription in low-income patients [63]. A systematic review assessing pharmacist-led deprescription initiatives demonstrated positive economic benefits but found limited impact on mortality, quality of life, falls, or hospitalizations [64]. In Canada, the D-PRESCRIBE study exemplified how pharmacist-led deprescription efforts reduced sedative medication use among the elderly, resulting in a 43 percent reduction over six months [65]. In Japan, pharmacists’ interventions in managing polypharmacy among cancer patients using opioids have been pivotal [66].

Community pharmacists dispense medications, provide guidance on side effects and interactions, verify prescription accuracy, and offer health advice, disease prevention strategies, and lifestyle recommendations. Additional services include vaccinations, medication initiation, blood sugar and blood pressure measurements, and mole scanning [67]. We designed this study to explore the feasibility of implementing NSAIDs deprescription in community pharmacies within the Norwegian healthcare system context. The specific objectives were: (1) to assess community pharmacists’ self-confidence for NSAIDs deprescription as part of their practice; (2) to understand community pharmacists’ attitudes related to deprescription as a service; and (3) to explore the requirements for introducing such an initiative in community pharmacies in Norway, considering opportunities and obstacles. 

## 2. Methods

### 2.1. Study Design and Target Group

A cross-sectional study was conducted to explore community pharmacists’ opinions, attitudes, and perceptions regarding their potential role in the deprescribing of NSAIDs in Norway. The study encompassed all community pharmacists across Norway (with either a bachelor’s or master’s degree in pharmacy), irrespective of age, gender, or geographical location. It is important to note that in Norway, individuals with a Bachelor of Pharmacy degree (or higher, i.e., Master of Pharmacy) are called community pharmacists, while pharmacy technicians, who have completed vocational education, cannot be called pharmacists. There is a clear distinction between these two roles. Community pharmacists must have at least a Bachelor of Pharmacy with 3 years of university education and are responsible for dispensing medications, providing patient counseling, and offering health advice in community pharmacy settings. They have the legal authority to counsel patients on medication use, perform some clinical tasks, and ensure the safe and effective use of pharmaceuticals. Pharmacy technicians take a Vocational Education in Pharmacy Technology and assist pharmacists by preparing medications, managing inventory, performing administrative tasks, and providing customer service. They work under the supervision of a licensed pharmacist and do not have the authority to perform clinical tasks or provide independent patient counseling. Therefore, in this study, we only targeted community pharmacists, who must have at least a bachelor of pharmacy degree with counseling tasks independently. Pharmacy technicians who do not have the same level of responsibility or authority as pharmacists were not included. In addition, due to the purpose of this study and the study focus, hospital pharmacists were not included. Hospital pharmacists in Norway work within hospital settings, collaborating with healthcare teams to optimize medication use, ensuring the safe administration of drugs, and providing direct patient care in clinical settings. They may also be involved in clinical trials and pharmaceutical research. 

Data collection utilized a digital questionnaire in Norwegian, chosen for its efficiency in gathering comprehensive information. Recruitment began on 9 January 2024 and concluded on 30 January 2024, allowing participants ample time to respond.

### 2.2. Survey

The finalized questionnaire drew inspiration from key articles identified in the existing literature [11,68,69,70,71,72,73] (Supplementary Material S1). The questionnaire was semi-structured, combining closed-ended and open-ended questions to provide both quantitative and qualitative insights. Distributed via the “Farmasi” closed Facebook group, which includes approximately 5800 pharmacist members, the questionnaire aimed to gather diverse perspectives. Please note that the closed Facebook group “Farmasi” in Norway is primarily intended for community pharmacists. However, it is inclusive of various types of pharmacists, including those working in hospitals. This group aims to foster communication and collaboration among pharmacists across different settings to improve professional practices and patient care. We only targeted community pharmacists within this closed Facebook group. 

#### 2.2.1. Pilot Testing

Before full-scale recruitment, a pilot test involving seven pharmacists ensured the questionnaire’s clarity, structure, and relevance. Feedback from participants guided minor adjustments to enhance comprehensibility.

#### 2.2.2. Content of the Questionnaire

The questionnaire comprised 35 questions, predominantly closed-ended with two optional open-ended comment fields. It was divided into five parts:

Part 1: Sociodemographic characteristics of participants

Part 1 of the questionnaire aimed to gather essential sociodemographic information from participants. It included six basic questions, with additional options for supplementary questions to ensure comprehensive data collection. The gathered information encompassed gender, age group, work experience, level of education, and geographical location of participants’ workplaces in Norway. These data were crucial for correlating sociodemographic factors with respondents’ views on deprescription, providing a holistic understanding of the study population.

A pivotal question ensuring relevance was: “Do you work as a pharmacist in a pharmacy (chain pharmacy or private pharmacy)?” This filter ensured that responses were from community pharmacists, aligning with the study’s focus on this specific professional group.

Part 2: Knowledge of deprescription

Part 2 consisted of six questions designed to gauge respondents’ basic knowledge of deprescription. Participants were asked to categorize statements as “Correct”, “Wrong”, or “Don’t know”, aiming to assess foundational understanding rather than in-depth expertise.

Questions covered fundamental aspects of deprescription, such as its definition, purpose, and procedural aspects. This approach allowed quick identification of general knowledge gaps among community pharmacists, essential for future educational initiatives and for promoting safe medication practices.

Part 3: Confidence in implementing deprescription of NSAIDs

Part 3 focused on evaluating pharmacists’ confidence in implementing deprescription of NSAIDs in their practice. It included five statements regarding their competence and readiness, rated on a scale from “Strongly agree” to “Strongly disagree”.

This section aimed to uncover pharmacists’ comfort levels in identifying appropriate deprescription scenarios, their initiative in suggesting dosage changes, and their confidence in managing NSAIDs-related adverse events and interactions. It also assessed perceptions of the relevance and effectiveness of their education in preparing them for deprescription discussions with colleagues and patients.

Part 4: Attitudes toward deprescription

Part 4 explored pharmacists’ attitudes toward deprescription through five statements, asking respondents to indicate their level of agreement from “Strongly agree” to “Strongly disagree”.

The section probed beliefs on deprescription’s potential benefits in reducing side effects and improving adherence, the feasibility of identifying deprescription opportunities during reviews, and the prioritization of deprescription in training needs. It encouraged reflections on the pharmacist’s role in deprescription and their impact during medication reviews.

Part 5: Challenges and opportunities for deprescription implementation

Part 5 focused on assessing challenges and opportunities for implementing deprescription in practice. Participants rated seven factors affecting deprescription on a Likert scale from 1 to 5, with 5 indicating the highest obstacle.

This section quantified perceptions of barriers such as time constraints, financial considerations, knowledge gaps, concerns about negative outcomes post deprescription, communication difficulties with prescribers, and patient or family resistance. It provided insights into operational challenges pharmacists face and identified areas needing additional support or resources for safe and effective medication management.

The concluding section invited further comments, allowing respondents to share additional insights or concerns not covered by predefined questions, ensuring comprehensive data collection.

These structured sections collectively aimed to gather nuanced insights into community pharmacists’ readiness, attitudes, and challenges regarding the deprescription of NSAIDs in Norway. Participants were informed of the study’s purpose, anonymity, and voluntary participation at the outset. Consent was implicit upon beginning the questionnaire, permitting data use for academic purposes, including this master’s thesis and potential scientific publications. A time estimate for completing the questionnaire was provided, concluding with gratitude for the participants’ contributions.

### 2.3. Ethics

The survey prioritized anonymity and voluntary participation, with no collection of personal or sensitive data, thus obviating the need for formal ethical approval. However, as a precautionary measure, the survey was submitted to the Data Protection Services for Research in Norway, Norwegian Agency for Shared Services in Education and Research (SIKT) for informational review and feedback (case number: 918711). Following confirmation that the survey did not involve sensitive data, it was distributed. The original questionnaire (Norwegian) and its translated version (English) are available as Supplementary Material S2. 

### 2.4. Data Handling

Data collected via Nettskjema were downloaded as a Microsoft Excel (version 2402) file and imported into SPSS for statistical analysis (IBM^®^ SPSS^®^ Statistics version 29.0.1.0.). A minimum of 100 responses has been recommended to ensure an accepted statistical power [74].

### 2.5. Statistical Analysis

Statistical analysis involved several methods to test hypotheses derived from the questionnaire. Table 1 outlines the dependent and independent variables, alongside hypotheses tested at a significance level (α) of 0.05. To mitigate the risk of Type I errors due to multiple analyses, a Bonferroni correction adjusted the significance level to 0.0071 for multiple tests (test of 7 formulated hypotheses) on the same dataset (i.e., 0.05/7 = 0.0071) [74]. In cases where cell frequencies were low, the Fisher’s Exact Test was employed to ensure robustness, as it is more suitable than the Chi-square test for sparse data distributions [74]. The use of appropriate statistical methods tailored to the dataset’s characteristics enhances the reliability of findings, supporting valid conclusions drawn from the survey results.

## 3. Results

### 3.1. Response Rate

The survey received responses from 73 participants. With a total of 35 questions in the questionnaire, an expected response rate of at least five responses per question was initially anticipated (i.e., 35 × 5 = 175). The actual response rate was then determined according to this, i.e., 73/175 × 100 = 41.7%. It is notable that response rates for questionnaires typically range between 30% and 50% and recent studies have indicated that the average response rate for online questionnaires stands at approximately 44.1% [75]. Despite targeting a sample size from the Facebook page of the Norwegian pharmacists, which has 5800 members, we did not achieve our expected high participant numbers. These limitations should be considered when interpreting the study results and the subsequent analysis conducted.

### 3.2. Descriptive Statistics

#### 3.2.1. Sociodemographic Characteristics of Study Participants

Table 2 presents the sociodemographic profile of the 73 survey respondents, all of whom are pharmacists working or having worked in Norway. The majority (97.4%) hold a license or authorization to practice as pharmacists in Norway, and 94.8% are currently employed in pharmacies. Women constitute the majority of respondents (87.7%), reflecting the gender distribution within the profession.

Age distribution shows a diverse range, with 46.6% of respondents aged 32 and above, indicating a varied demographic. In terms of work experience, 32.9% have over 10 years of experience, highlighting a cohort with considerable professional tenure.

Regarding educational qualifications, 57.5% possess a master’s degree in pharmacy, underscoring the educational attainment within the respondent group. Geographically, a significant proportion (65.8%) work in Eastern Norway, aligning with population distribution trends.

Moreover, 89% of respondents received their pharmacy education in Norway, while the remaining 11% received education from various countries including Germany, Iraq, Sweden, Serbia, Moldova, and Ethiopia, showcasing the international diversity among pharmacy professionals in Norway. 

#### 3.2.2. Knowledge about Deprescription

In Part 2 of the survey, which assessed respondents’ knowledge of deprescription, clear trends emerged from the 73 participants’ responses. There was unanimous agreement among all participants that deprescription serves the purpose of reducing serious side effects, improving patient health, or minimizing unnecessary medication use. Similarly, a significant majority (97%) correctly identified deprescription as a planned process for stopping or reducing drug dosages.

However, the survey revealed varying levels of understanding of other aspects of deprescription. Only three respondents believed that deprescription should exclusively be initiated in response to side effects, contrasting with the majority opinion of 67 participants who found this statement incorrect. A substantial consensus (77%) emphasized that deprescription decisions should involve the patient, highlighting the importance of patient-centered care in this process. Additionally, opinions were divided regarding whether deprescription and discontinuation are synonymous concepts: 26% viewed them as identical, while 62% recognized distinctions between the two terms.

**Figure 1:**
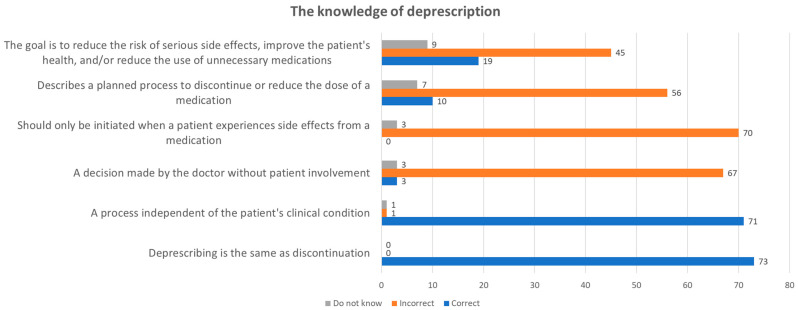
Summary of respondents’ knowledge about deprescription.

Figure 1 provides a visual summary of these insights.

#### 3.2.3. Confidence in Implementing Deprescription of NSAIDs in Practice

**Figure 2:**
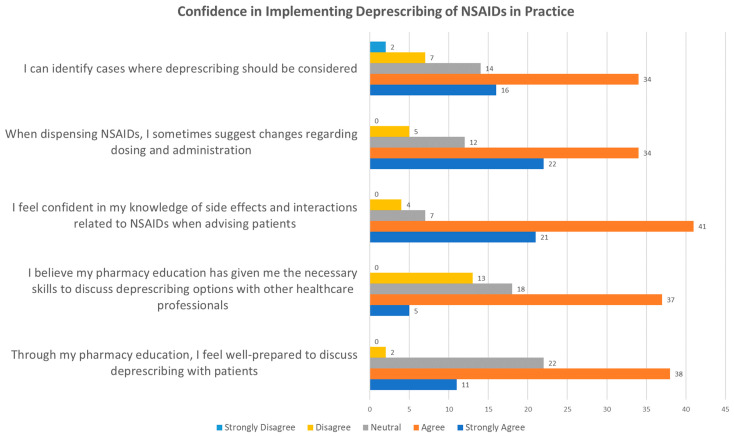
Respondents’ self-confidence related to the implementation of deprescription of NSAIDs in practice.

Figure 2 illustrates the respondents’ confidence in their ability to implement the deprescription of NSAIDs. Among the 73 participants, a majority (67%) expressed strong confidence in their ability to identify cases where NSAIDs deprescription should be considered, with 49 either agreeing or strongly agreeing. Similarly, a substantial number of respondents felt comfortable suggesting dosage and administration changes, although there was some level of uncertainty or disagreement.

Confidence in the knowledge of side effects and interactions related to NSAIDs was particularly high, indicating a strong sense of competence in this area among the respondents. This is crucial, as understanding adverse effects and interactions is vital for safe and effective deprescription practices.

Respondents generally viewed their education as adequate preparation for engaging in deprescription discussions with patients and other healthcare professionals. However, some participants indicated hesitation, suggesting that there might be room for improvement in pharmacy education to better equip pharmacists for these discussions.

#### 3.2.4. Attitudes Related to Deprescription

**Figure 3:**
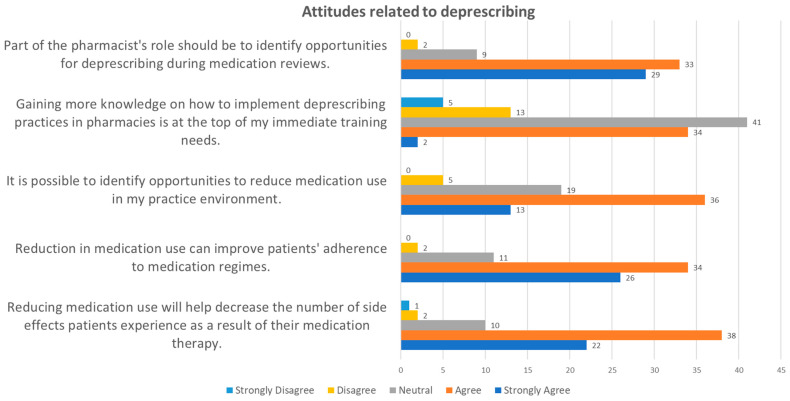
Respondents’ attitudes toward deprescription.

Part 4 of the survey focused on assessing community pharmacists’ attitudes toward deprescription. The responses revealed several key insights into their perspectives on reducing medication use, identifying opportunities for deprescription, and the role of pharmacists in this process (Figure 3).

More than half of the respondents strongly supported the idea that reducing medication use can contribute to fewer side effects and better adherence to medication regimens. This indicates a positive attitude toward the potential benefits of deprescription.

There was a consensus among respondents that pharmacists have a role in identifying deprescription options during medication reviews. This emphasizes the perceived importance of pharmacists in optimizing medication therapy through deprescription.

Despite the general support for deprescription, acquiring additional knowledge about deprescription was not considered a top priority by most respondents. This suggests that they may feel adequately prepared or have other pressing training needs within their practice.

Some respondents highlighted the lack of clear guidelines for the long-term use of NSAIDs, particularly when used in combination with other medications like blood thinners or antihypertensives. There was a noted desire for more training and emphasis on deprescription within pharmacy practices. Respondents emphasized the importance of tools such as STOPP-2 and NorGep criteria for comprehensive medication assessments. Specific concerns were raised about the duration of treatment with NSAIDs and the need for improved communication among healthcare professionals to effectively manage deprescription scenarios.

These insights suggest opportunities for enhancing pharmacist education and training programs to better support deprescription practices, ensuring safer and more effective medication management for patients.

#### 3.2.5. Challenges and Opportunities for Implementing Deprescription in Practice

In assessing the challenges related to implementing deprescription in practice, the survey highlighted various perceived obstacles among community pharmacists. Table 3 summarizes these challenges and the level of challenge based on self-reported perceptions of the respondents on a scale of 5, from minimal obstacles (1) to major (5).

The majority of respondents identified lack of time as a significant barrier to implementing deprescription. This suggests that time constraints in daily pharmacy practice may hinder thorough medication review and deprescription discussions. Concerns about financial compensation for reviewing drug use indicate that incentives could play a crucial role in optimizing deprescription processes. This highlights the importance of considering economic factors in promoting medication management practices. There is a recognized need for more extensive knowledge about tools and methods for deprescription among community pharmacists. This highlights opportunities for additional training and education to enhance proficiency in deprescription practices. It is conceivable that community pharmacists must undergo a course to become service pharmacists who can carry out drug withdrawal. In the same way, only pharmacists who have completed courses in vaccination, inhalation guidance, and medication initiation have received a diploma and can carry out these services. Apprehensions regarding potential negative outcomes after deprescription decisions were also prominent. Addressing these concerns through evidence-based guidelines and enhanced pharmacist education could mitigate perceived risks. Difficulty in communicating with prescribers and encountering resistance to recommendations were noted as barriers. Improving interprofessional communication and collaboration could facilitate smoother deprescription processes. While less frequently mentioned, reluctance from patients or their relatives was acknowledged as a factor influencing deprescription decisions. Strategies to improve patient education and engagement may help address this concern.

Respondents expressed concerns about inadequate access to complete patient records, which limits their ability to make informed deprescription decisions. Suggestions were made for a more integrated model where pharmacists collaborate closely with physicians and other healthcare professionals to optimize deprescription strategies. Some respondents highlighted the need for tailored deprescription approaches, particularly concerning NSAIDs and specific patient demographics. Challenges related to language barriers and the complexities of polypharmacy were also raised, underscoring the multifaceted nature of deprescription challenges.

### 3.3. Inferential Statistics

Inferential statistics were used to test several null hypotheses related to pharmacists’ perceptions and cooperation in the deprescription of NSAIDs. An overview of the results of the statistical analyses is summarized in Table 4.

### 3.4. Existing Guidelines 

The literature search findings revealed a notable emphasis on guidelines on opioid deprescription, with several documents specifically addressing this class of drugs. Noteworthy sources include guidelines from the Norwegian Directorate of Health and the National Institute for Health and Care Excellence (NICE), which offer comprehensive recommendations and strategies for the safe and effective deprescription of opioids in adult patients. 

In contrast, there is a scarcity of guidelines dedicated specifically to the deprescription of non-opioid medications, such as NSAIDs (nonsteroidal anti-inflammatory drugs). While some broader guidelines touch on aspects of deprescription and drug optimization, they do not specifically target NSAIDs. Relevant documents include reports from the Department of Health & Social Care and technical guidelines from the World Health Organization (WHO).

The implications of these findings for both practice and research are multifaceted. First, they stress the necessity for further development of policies and recommendations that address the deprescription of non-opioids, particularly considering the pivotal role of community pharmacists in this process. Second, the existing guidelines highlight the critical importance of integrating deprescription strategies into clinical practice to enhance patient safety and treatment outcomes.

## 4. Discussion

This research project focused on exploring the opportunities and challenges associated with the deprescription of non-opioid analgesics, specifically NSAIDs, within pharmaceutical practice in Norway. A critical analysis of existing literature identified a significant knowledge gap regarding the roles and obstacles faced by community pharmacists in this context. To address this gap, a survey was conducted to investigate pharmacists’ perspectives, attitudes, and self-confidence concerning NSAIDs deprescription.

The study revealed that community pharmacists exhibit a high level of confidence in recognizing situations where NSAIDs deprescription is appropriate. However, they also encounter substantial challenges. These challenges include time constraints, lack of financial incentives, and communication barriers with prescribers, collectively hindering the implementation of deprescription practices. Moreover, the survey highlighted a notable absence of specific guidelines and training tailored for NSAIDs deprescription. This underlines the urgent need for the development of comprehensive guidelines to support pharmacists in this critical area of practice.

Interestingly, the findings also indicated a positive attitude among community pharmacists toward deprescription. They recognize that reducing medication can mitigate side effects and enhance patient adherence to treatment regimens. This positive stance suggests a promising potential for expanding deprescription practices within pharmacy settings, contingent upon addressing the aforementioned challenges effectively.

### 4.1. Discussion of Method

#### 4.1.1. Choice of Method

This study employed a cross-sectional survey to capture the perceptions and experiences of pharmacists across Norway regarding NSAIDs deprescription. While effective in gathering a wide range of responses within a short timeframe, this design has inherent limitations. Notably, it does not establish causal relationships between pharmacists’ perceptions and their actual practices. For instance, while the survey revealed a correlation between perceived time constraints and challenges in deprescription, it cannot conclusively determine whether time scarcity directly causes these challenges or if other factors are also influential.

Moreover, the cross-sectional nature of the study provides a snapshot of current perceptions and practices, lacking insights into longitudinal changes over time. This aspect is crucial given the dynamic nature of healthcare practices, where evolving policies and training initiatives could potentially influence pharmacists’ approaches to deprescription.

The decision to employ a questionnaire alongside a literature search was deliberate, driven by practical considerations and the study’s specific research context. Questionnaires, particularly those administered digitally, offer efficiency and scope advantages over qualitative methods like interviews. They facilitate broader geographic coverage, anonymity, and potentially higher response rates. These factors are crucial in engaging a diverse and representative sample of pharmacists.

While the survey yielded insights from 73 respondents, providing initial perspectives on pharmacists’ attitudes toward deprescription, the relatively small sample size limits the study’s statistical power and generalizability. Additionally, the overrepresentation of respondents from Eastern Norway introduces a potential selection bias, impacting the study’s external validity due to regional variations in healthcare practices and resources.

#### 4.1.2. Integration of Qualitative Data

Although primarily quantitative, the study incorporated open comment boxes in the questionnaire to capture qualitative insights. This approach allowed respondents to elaborate on their responses, providing nuanced perspectives that enriched the data. However, it should be noted that this method does not replace the depth of traditional qualitative techniques like in-depth interviews, yet it adds flexibility and depth to the findings.

### 4.2. Discussion of the Results

#### 4.2.1. Response Rate

The study achieved a response rate of 41.7%, with 73 pharmacists participating, placing it within the typical range observed for digital surveys, as documented in educational research meta-analyses [75]. This rate reflects persistent challenges in digital survey participation due to factors like survey fatigue and digital disruptions. Future surveys could explore alternative distribution methods, such as collaboration with pharmaceutical unions, to enhance targeted outreach and improve response rates.

#### 4.2.2. Results from Testing Hypotheses and Sociodemographic Characteristics

Gender Distribution: The study showed a predominant representation of females (87.7%), consistent with national figures for the pharmaceutical profession. Statistical tests revealed no significant gender-based differences in perceptions related to reluctance toward deprescription among patients or prescribers’ receptivity to recommendations.

Age and Experience: Respondents exhibited a diverse age distribution, with a notable proportion (46.6%) aged over 32 years, indicating a mix of both early career and experienced pharmacists. Experience level (over 10 years for 32.9% of respondents) did not significantly correlate with attitudes toward integrating NSAIDs deprescription into medication reviews.

Education Level: A majority (57.5%) of respondents held a master’s degree in pharmacy, reflecting high educational standards among Norwegian pharmacists. The study found no significant association between education level (bachelor’s vs. master’s) and pharmacists’ competence in identifying cases of deprescription in patient interactions.

Geographical Distribution: Most respondents (65.8%) worked in Eastern Norway, highlighting potential geographical bias. Despite this, there was no statistically significant correlation between geographical location and perceived barriers such as lack of time for deprescription, suggesting universal challenges across regions.

Education Origin: The majority (89%) of respondents received their pharmacy education in Norway. The analysis did not show a significant link between Norwegian education and pharmacists’ confidence in discussing deprescription with patients. This marks the ongoing need for educational programs that prepare pharmacists uniformly for deprescription challenges.

#### 4.2.3. Knowledge about Deprescription

Respondents generally demonstrated a solid understanding of deprescription, recognizing it as a planned process to reduce side effects and unnecessary medication use. However, misconceptions surfaced, such as the belief that deprescription should only be initiated in response to side effects, and confusion between deprescription and discontinuation. These findings call attention to the importance of targeted education to clarify concepts and emphasize patient-centered approaches in deprescription decisions.

The study aligns with international research highlighting pharmacists’ comprehensive knowledge of deprescription [70]. This reinforces the call for enhanced training initiatives, potentially leveraging existing resources like e-learning platforms, to bolster pharmacists’ confidence and competence in deprescription practices.

#### 4.2.4. Pharmacists’ Confidence in Implementing Deprescription in Practice

The discussion on pharmacists’ confidence in implementing deprescription reveals crucial insights into their role and competence in medication management, particularly concerning NSAIDs. The survey results highlight pharmacists’ perceptions of their own abilities and confidence levels related to deprescription, shedding light on their readiness to engage in this complex aspect of pharmaceutical practice.

A significant majority of respondents demonstrate strong agreement in their ability to identify cases where deprescription of NSAIDs is appropriate. This high level of confidence points to pharmacists’ pivotal role in recognizing situations where reducing or discontinuing NSAIDs use can enhance patient safety and optimize therapeutic outcomes. Similar studies conducted internationally, such as in Ireland and Qatar, also reflect pharmacists’ robust confidence in managing NSAIDs-related issues, emphasizing a global recognition of their competence in deprescription processes [69,70]. This confidence is particularly crucial amid concerns over medication overuse, highlighting pharmacists’ potential to mitigate adverse effects, drug interactions, and unnecessary healthcare costs, thereby contributing to a more sustainable healthcare system.

Moreover, a substantial proportion of pharmacists express comfort in recommending dosage and administration changes when dispensing NSAIDs. While some respondents indicate neutrality or disagreement, the overall positive response suggests that many pharmacists are actively implementing deprescription principles in their daily practice. This aligns with research indicating pharmacists’ extensive knowledge of NSAIDs side effects and their proactive role in educating patients about safe medication use, underscoring their impact on promoting informed decision-making and patient safety [69].

Pharmacists’ confidence in their knowledge of NSAIDs side effects and interactions, as observed in the survey, further supports their ability to provide comprehensive guidance to patients. Effective patient counseling hinges on pharmacists’ deep understanding of these critical aspects, enabling them to advise on optimal medication use and potential deprescription strategies where applicable.

While respondents generally report satisfaction with their pharmacy education regarding deprescription discussions, a notable proportion expresses less confidence. This highlights a clear need for ongoing professional development and tailored training initiatives to bolster pharmacists’ skills in navigating deprescription conversations effectively. Strengthening regulatory frameworks and guidelines specific to deprescription could empower pharmacists to confidently utilize their expertise and authority in patient interactions, ensuring consistent practices across healthcare settings.

#### 4.2.5. Attitudes Related to Deprescription

The survey results highlight pharmacists’ attitudes toward deprescription, revealing a positive stance toward its role in improving patient safety and medication management.

A majority of respondents strongly agree or agree that reducing drug use can mitigate side effects experienced by patients. This consensus makes evident pharmacists’ recognition of deprescription as a critical strategy to minimize adverse drug reactions, particularly amidst concerns surrounding polypharmacy.

Similarly, pharmacists acknowledge that reducing medication can enhance patient adherence to treatment regimens. This reflects an understanding of deprescription’s potential to optimize therapeutic outcomes by promoting medication adherence and minimizing unnecessary drug use.

While many pharmacists recognize opportunities for deprescription in practice, challenges persist in implementing these practices effectively. Factors such as resource constraints, inadequate training, or support systems may hinder pharmacists from identifying and executing deprescription strategies optimally.

Interest in further education on deprescription appears moderate, with a sizable portion of respondents expressing neutrality. This suggests varying perceptions among pharmacists regarding their current knowledge levels and training needs in deprescription practices.

A significant proportion of respondents agree that identifying opportunities for deprescription should be integral to pharmacists’ roles during medication reviews. However, concerns about time constraints highlight the perceived challenge of integrating deprescription activities into routine pharmacy workflows.

Overall, the findings indicate a positive attitude toward deprescription among pharmacists, coupled with a recognition of the need for enhanced training and resources to support its effective implementation. Beyond pharmaceutical practice, these attitudes highlight the broader societal benefits of improving health literacy and promoting informed decision-making regarding rational pharmacotherapy, particularly in pain management contexts.

#### 4.2.6. Challenges and Opportunities for Implementing Deprescription in Practice

The discussion on challenges and opportunities associated with deprescription implementation provides critical insights into the practical barriers faced by pharmacists in this domain.

Over 67% of respondents identify time constraints as a significant or primary obstacle to considering deprescription prescriptions. This highlights the demanding nature of pharmacists’ current responsibilities and the need for dedicated time and resources to facilitate deprescription initiatives effectively. Many respondents cite the lack of financial incentives for reviewing drug use as a notable challenge. Establishing financial incentives could motivate pharmacists to engage more actively in deprescription activities, similar to existing models for managing other chronic conditions. Although less prevalent, concerns about potential negative consequences following deprescription decisions indicate a degree of uncertainty among pharmacists. Addressing these concerns through comprehensive training and evidence-based guidelines can help mitigate fears and build confidence in deprescription practices.

Effective communication with prescribers and their receptivity to pharmacists’ recommendations are critical for successful deprescription outcomes. Improved communication channels and mutual recognition of pharmacists’ expertise are essential to overcoming these challenges and fostering collaborative relationships in patient care.

While not the primary obstacle for most respondents, reluctance from patients or their relatives marks the importance of effective patient education and communication about the benefits of deprescription. Addressing misconceptions and discussing alternative treatment options can facilitate informed decision-making and support patient acceptance of deprescription strategies.

Open comment feedback highlights the need for comprehensive patient information and better integration of pharmacists into clinical care teams. Enhancing access to patient data and improving interdisciplinary collaboration can address barriers such as limited patient insight and facilitate follow-up on deprescription outcomes.

These insights demonstrate the multifaceted challenges associated with deprescription implementation in practice and advocate for targeted interventions to support pharmacists. Enhancing training programs, securing financial and institutional support, and strengthening collaboration across healthcare disciplines are essential steps toward empowering pharmacists to play a more proactive and effective role in deprescription initiatives. By addressing these challenges, healthcare systems can optimize medication management, enhance patient safety, and promote sustainable healthcare practices.

#### 4.2.7. Guidelines for Deprescription Analgesics

A literature search conducted as part of this study provides valuable insights into the current landscape of guidelines for deprescription analgesics, particularly concerning the involvement of community pharmacists. While the search identified a limited number of relevant documents, it shows a critical area for future research and policy development: the need for more detailed and specific guidelines that delineate the role of community pharmacists in the deprescription process.

Out of the initial 1735 documents screened, only 12 were considered relevant following a rigorous selection process. This highlights both the stringent criteria applied and the potential deficiency in existing guidelines specifically addressing the role of community pharmacists in deprescription, particularly for non-opioid analgesics. This scarcity highlights an opportunity for future research to bridge this gap by developing guidelines tailored to integrate pharmacists’ expertise and resources into deprescription processes effectively.

While general guidelines for medication review and management exist, the findings emphasize the necessity to specifically address and enhance the role of community pharmacists in deprescription. This includes activities ranging from identifying deprescription candidates to providing ongoing support throughout the deprescription journey. Clear directives and recommendations targeted at these specific tasks are essential to optimize the safe and effective implementation of deprescription strategies.

The absence of directly relevant national documents in Norway, despite some pertinent European guidelines, urges immediate attention to an opportunity for Norwegian health services to lead in the development of innovative and context-specific guidelines for deprescribing analgesics. This initiative could significantly advance pharmacists’ involvement in deprescription practices and set a precedent for other healthcare systems to follow suit.

The literature search findings advocate for further research and policy development aimed at bolstering the role of community pharmacists in deprescription processes. By addressing this gap, policymakers and healthcare providers can promote the formulation of more precise, targeted guidelines that enhance patient outcomes by reducing unnecessary medication use and improving therapeutic appropriateness.

This observation resonates with previous studies that have also identified a dearth of clinical guidelines for deprescription, particularly among older populations. Addressing this gap through tailored guidelines can facilitate the integration of pharmacists’ expertise into deprescription practices and align with the broader goal of optimizing medication management across healthcare settings.

In short, the findings from the literature search highlight a clear need for enhanced guidelines tailored to empower community pharmacists in the deprescription of analgesics. By advancing this agenda, healthcare systems can leverage pharmacists’ roles more effectively to mitigate polypharmacy risks, enhance patient safety, and promote sustainable healthcare practices.

### 4.3. Strengths and Weaknesses of the Study

In designing this study on the role of community pharmacists in deprescribing NSAIDs, careful attention was given to methodological rigor to ensure reliability and validity. As mentioned earlier in the discussion of the method (Section 4.1), there are a number of ways for improvement. Since no standard questionnaire was available, a survey was constructed, and the pilot test involving seven respondents provided valuable feedback on questionnaire clarity and content refinement before the final distribution. This process can be improved by following multiple steps proposed for the construction of a questionnaire [76]. The low response rate from the target group of community pharmacists in Norway is an important limitation that also influences the generalizability of the findings. Future studies should include a broader area and a larger sample size to determine if the results of this study can be substantiated. Future studies must also adapt qualitative designs (e.g., interviews) to deepen the results of the quantitative survey. 

## 5. Conclusions

The study revealed that community pharmacists generally exhibited high confidence in identifying the need for deprescribing NSAIDs and expressed optimism about its implementation in their pharmacy practice. However, significant barriers such as time constraints, lack of financial compensation, and communication challenges with prescribers were highlighted as potential barriers. Respondents acknowledged the critical importance of accessing patient information to overcome barriers arising from inadequate insight into patients’ health status. To promote safer and more rational use of NSAIDs, coordinated cooperation among health authorities, educational institutions, and healthcare professionals was recommended. Additionally, the development and implementation of detailed policies and training programs specifically tailored to NSAIDs deprescription were emphasized. Establishing financial incentives and supportive structures that recognized the time and effort pharmacists invested in deprescription initiatives was also deemed crucial. Strengthening communication channels between pharmacists, patients, and prescribers was also named as essential to foster better collaboration and deepen understanding of the importance of deprescription.

## Figures and Tables

**Table 1:** Overview of the null hypotheses.

Null Hypothesis	Independent Variable	Variable, Measured Value	Dependent Variable	Variable, Measured Value	Statistical Analysis
There is no significant association between work experience and whether respondents report that deprescription of NSAIDs should be included in the medication review.	Work experience	Continuous	Deprescription should take place during a medication review	Categorical, ordinal	Ordinary regression
There is no significant difference between women and men in their perception that there may be reluctance on the part of the patient or their relatives to implement deprescription.	Gender	Categorical, nominal	The perception that there may be reluctance from the patient or relatives to implement deprescription	Categorical, ordinal	Chi-square test
There is no correlation between the level of education and pharmacists’ ability to identify cases where deprescription should be considered.	Level of education	Categorical, ordinal	The ability to identify cases for deprescription	Categorical, ordinal	Chi-square test
There is no correlation between age and perception of communication and the availability of prescribers as a barrier.	Age	Categorical, ordinal	Perception of communication and accessibility to prescribers a barrier	Categorical, ordinal	Chi-square test
There is no correlation between the place of work and the perception of lack of time as a barrier.	Location	Categorical, nominal	Lack of time	Categorical, ordinal	Chi-square test
There is no correlation between gender and those who believe that prescribers are not very receptive to recommendations.	Gender	Categorical, nominal	The perception that prescribers are not very receptive to recommendations	Categorical, ordinal	Chi-square test
There is no correlation between having a pharmacy degree from Norway and reported confidence in discussing deprescription with patients.	Pharmacy education in Norway	Categorical, dichotomous	Confidence to discuss deprescription with patients	Categorical, nominal	Chi-square test

**Table 2:** Overview of results from sociodemographic characteristics of the study participants.

Question	Description	Number	Percent
Gender			
	Man	8	11.0%
	Woman	64	87.7%
	Other	1	1.4%
	Does not want to answer	0	0.0%
Age (years)			
	21–26	22	30.1%
	27–32	17	23.3%
	>32	34	46.6%
Work experience (years)			
	Newly qualified pharmacist (0–1)	17	24.7%
	<5	19	26.0%
	5–10	12	16.4%
	>10	24	32.9%
Level of education			
	Bachelor in Pharmacy	27	37.0%
	Master in Pharmacy	42	57.5%
	Other	4	5.5%
Place of work in Norway			
	Northern Norway	8	11.0%
	Central Norway	7	9.5%
	Western Norway	7	9.6%
	Eastern Norway	48	65.8%
	Southern Norway	3	4.1%
Pharmacy education obtained in Norway			
	Yes	65	89.0%
	No	8	11.0%

**Table 3:** Challenges for community pharmacists in implementing deprescription in practice. Please note that the challenges are shown on a scale of 1 to 5, where 1 signifies minimal challenges and 5 signifies the major obstacles perceived.

#	Challenge	Number of Responses	Mean	Median	Minimal (1)	Small (2)	Moderate (3)	Large (4)	Major (5)
1	Lack of time—not enough time to assess prescriptions for deprescription options	73	3.92	4	0	8	16	23	26
2	Lack of financial compensation for review of drug use	73	3.68	4	2	11	20	15	25
3	Lack of knowledge about tools and methods for deprescription	73	3.42	4	6	9	20	24	14
4	Concerns related to negative consequences after performing deprescription	73	3.19	3	5	13	27	19	9
5	Communication and the availability of prescribers are problematic	73	4.15	4	1	1	11	33	27
6	Prescribers are not very receptive to recommendations	73	3.58	4	0	5	30	21	17
7	Reluctance from the patient or relatives	73	3.33	3	2	10	28	28	5

**Table 4:** A summary of the results from testing the null hypotheses.

Null Hypothesis	Statistical Analysis	*p*-value	Null Hypothesis Discarded/Retain	Result
There is no significant association between work experience and opinions that deprescription of NSAIDs should be included in the medication review	Ordinary regression	0.988	Retained	The analysis revealed no significant correlation between work experience and attitudes toward the inclusion of deprescription of NSAIDs in medication reviews, which is reflected in a *p*-value of 0.988 from logistic regression analysis.
There is no significant difference between women and men in their perception that there may be reluctance from the patient or relatives to implement deprescription	Chi-square test	0.240	Retained	The Chi-square test showed no significant gender differences in perception of resistance to deprescription among patients or relatives, with a *p*-value of 0.240. Confirmatory analyses, including the Likelihood Ratio test (*p* = 0.228) and the Fisher’s Exact Test (two-sided *p* = 0.287; unilateral *p* = 0.215), also underlined the absence of significant differences.
There is no correlation between the level of education and community pharmacists’ ability to identify cases where deprescription should be considered	Chi-square test	0.954	Retained	The results showed that there is no statistically significant association (Pearson Chi-Square = 1.582, df = 6, *p* = 0.954). This is confirmed by the Likelihood Ratio (*p* = 0.909) and the Fisher–Freeman–Halton Exact Test (*p* = 0.969), both of which support the null hypothesis.
There is no correlation between age and perception of communication and availability of prescribers as a barrier	Chi-square test	0.498	Retained	The Chi-Square test indicated no statistically significant difference in the perception of communication and accessibility to prescribers as a barrier based on age, with a *p*-value of 0.498. The Likelihood Ratio test confirmed this finding with a *p*-value of 0.437. Due to a low number of expected observations in several cells, a Fisher–Freeman–Halton Exact Test was also used, which further supported the null hypothesis with a *p*-value of 0.459.
There is no correlation between the place of work and the perception of lack of time as a barrier	Chi-square test	0.935	Retained	In this study, the Chi-square test revealed no statistically significant correlation between the place of work and the perception of lack of time as a barrier in deprescription, with a *p*-value of 0.935. The Likelihood Ratio test and the Fisher–Freeman–Halton Exact Test, with *p*-values of 0.855 and 0.925, respectively, confirmed the absence of a significant difference.
There is no correlation between gender and those who believe that prescribers are not very receptive to recommendations	Chi-square test	0.522	Retained	The analysis revealed no statistically significant association between gender and the perception that prescribers are not very receptive to recommendations, with a *p*-value of 0.522. Confirmatory results from the Likelihood Ratio test (*p* = 0.431) and the Fisher–Freeman–Halton Exact Test (*p* = 0.756) also supported the null hypothesis.
There is no correlation between having a pharmacy degree from Norway and reported confidence in discussing deprescription with patients	Chi-square test	0.890	Retained	Analysis of the relationship between pharmacy education in Norway and self-confidence in discussing deprescription with patients revealed no statistically significant correlation, as shown by the Chi-square test and the Fisher–Freeman–Halton exact test, with *p*-values of 0.890 and 0.731, respectively. The use of the Fisher–Freeman–Halton exact test was particularly justified given that over 20% of the cells in the cross-table had expected frequencies below five, which could compromise the reliability of the Chi-square test, especially with a small sample size.

## Data Availability

All original data are available.

## Supplementary Materials

The following supporting information can be downloaded at: https://www.mdpi.com/article/10.3390/pharmacy12040116/s1, The literature search results (Supplementary Material S1); The questionnaire in Norwegian and English translation (Supplementary Material S2).
