# Supplementary material for: Deprescribing NSAIDs: The Potential Role of Community Pharmacists
Source: Pharmacy (Basel). 2024 Jul 24;12(4):116. doi: 10.3390/pharmacy12040116 (PMC11358956; doi:10.3390/pharmacy12040116)
Supplement: Supplementary file 1 [file pharmacy-12-00116-s001.zip › Supplementary Material 1.pdf]

Supplementary Material 1

Literature Search

This document provides a comprehensive overview of the literature and details the systematic literature search conducted to identify the most relevant articles for inclusion in the design of our questionnaire. This section shows in detail the specific steps and procedures followed, as well as the results obtained from the search process.

Research Question

Deprescribing is an important process to discontinue or reduce medications that are no longer deemed necessary or that may be harmful. This study aimed to explore and describe the possible roles of community pharmacists in the deprescribing process of both prescription and over-the-counter NSAIDs in Norway, a key factor in managing polypharmacy and improving patient outcomes in pain care. Based on this, the research question was formulated: "What opportunities and challenges do community pharmacists face in deprescribing prescription and over-the-counter NSAIDs in Norway?"

Search Strategy

In this study, the PICO model was used as a basis to develop the search strategy and identify the most relevant articles for our research purpose. PICO stands for Population (P), Intervention (I), Comparison (C), and Outcome (O). This framework helped us structure our literature search in a way that ensured we focused on studies directly relevant to our research area. **Table 1** shows an overview of how the PICO model was applied, with the research question as the basis for the design.

Table 1: Overview of how each component of PICO was defined and used to guide the search process

PICO Elements	Description
Population (P)	Community pharmacists working in both pharmacy chains and private pharmacies
Intervention (I)	Opportunities and challenges related to deprescribing NSAIDs
Comparison (C)	Not applicable
Outcome (O)	Rational pharmacotherapy, improved quality of life, fewer hospital admissions, patient safety, and improved pain therapy

Inclusion Criteria

The inclusion criteria were developed in collaboration between the master student (DA) and the supervisor (PG). The following criteria were carefully followed to conduct a systematic literature search, aiming to identify relevant articles for the design of our questionnaire.

- Articles addressing community pharmacists' views (experiences and attitudes) on deprescribing NSAIDs.
- Articles published in English and Norwegian.
- Only full-text articles.
- Articles published in the last 10 years.
- Qualitative or quantitative articles as research methods.

Exclusion Criteria

Exclusion criteria were developed alongside the inclusion criteria to ensure a focused and relevant selection of articles. The following types of articles were excluded from our study:

- Studies primarily concern hospital or clinical pharmacists, as our research is aimed at community pharmacists.
- Conference articles, opinion articles, case reports, and other non-empirical studies to ensure our analysis is based on robust empirical data.

Database

Databases used:

- PubMed
- Embase (Ovid)
- Web of Science

Search Strategy in Practice and PRISMA Diagram

To implement our search strategy in practice, the PICO framework was concretized into specific search terms and phrases. These search terms were carefully selected to reflect the core elements of the research question and were adapted for optimal use in the different databases: PubMed, Embase (Ovid), and Web of Science. The search strategy used in these databases was with the aid of search words, for example: "Community Pharmacist*" OR "Retail Pharmacist*" AND Deprescri* in PubMed and "Community Pharmacists" OR "Community pharmacy professionals" OR "Retail pharmacist" OR "Primary care pharmacist" AND Deprescri* OR "Drug tapering" OR "Drug Discontinuation" OR "Medication Withdrawal" OR NSAIDs OR "Non-Steroidal Anti-Inflammatory Drugs" in Web of Science.

The search in Embase (Ovid) was conducted on 30.11.23 and resulted in a total of 114 articles. The PubMed search was then conducted on 02.12.23, yielding 18 articles. Furthermore, the search in Web of Science, conducted on 04.12.23, resulted in 62 articles, further contributing to the breadth of our literature collection.

These 177 articles were carefully reviewed and evaluated according to our inclusion and exclusion criteria to ensure that only the most relevant and qualitative articles were selected for further analysis. It is important to note that the entire search and selection process was carried out by only one person, the researcher. To ensure high quality and transparency in this process, we based it on the PRISMA (Preferred Reporting Items for Systematic Reviews and Meta-Analyses) flow diagram. PRISMA is a recognized standard for reporting systematic literature reviews and meta-analyses. The entire search and selection process is detailed in Figure 1, which shows the step-by-step process from the original 194 articles to the 6 finally included and used to design our questionnaire.

Figure 1: PRISMA flow diagram

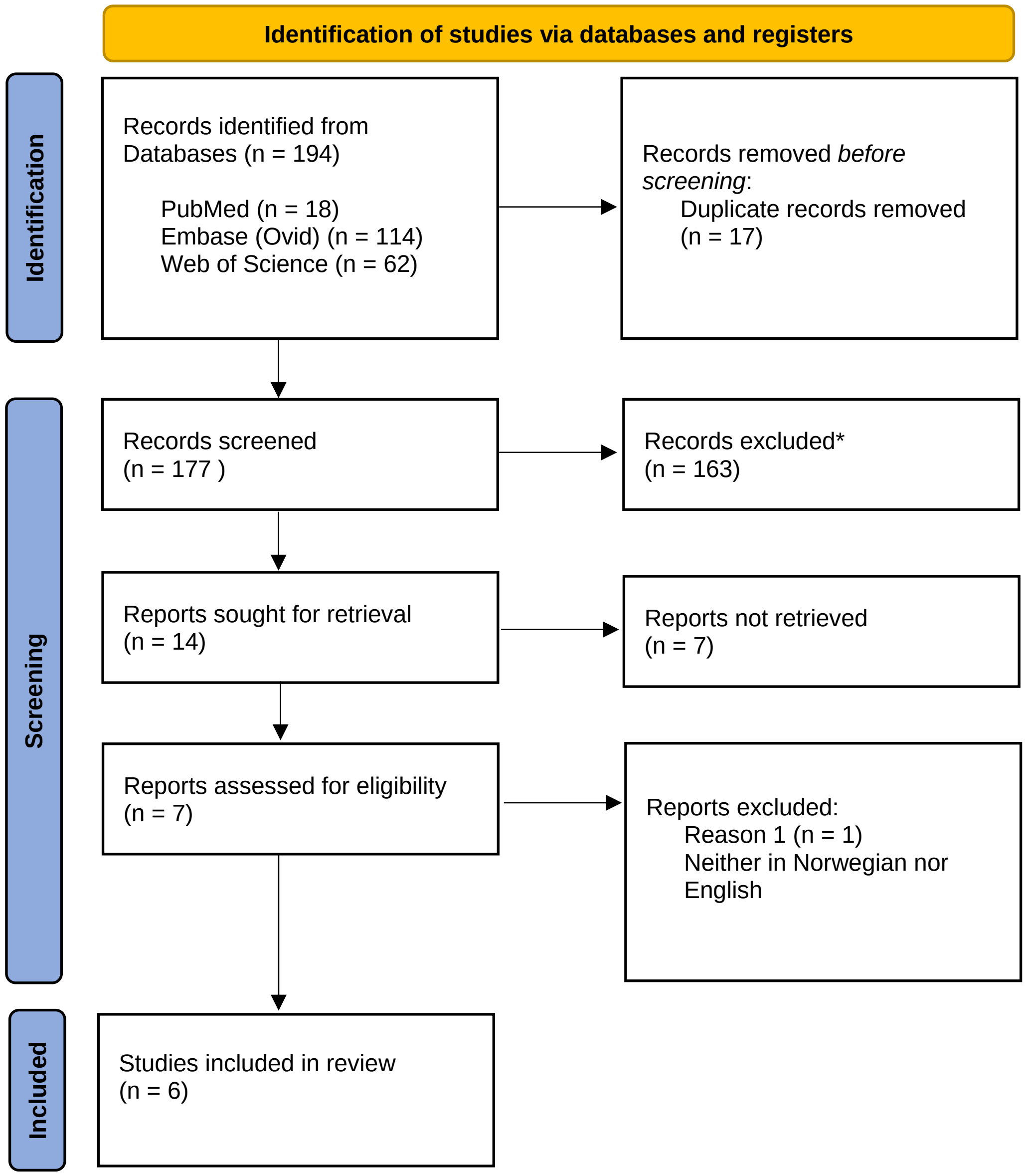

* Reason for exclusion:

- Not dealing with community pharmacists' views on deprescribing NSAIDs (n = 140)
- Neither qualitative nor quantitative research method (n = 19)
- Conference articles, opinion articles, case reports (n = 4)

Table 4 shows the included articles with information on the research method, purpose, study design, target group, main findings, and conclusion.

Table 4: Detailed overview of articles included in the design of the questionnaire

Article	Research Method	Purpose	Study Design	Target Group	Main Findings	Conclusion
Jawza Meaadi, Ilona Obara, Hamde Nazar, A qualitative study to investigate community pharmacists' perceptions about identifying and addressing inappropriately prescribed analgesia, International Journal of Pharmacy Practice, Volume 31, Issue 4, August 2023, Pages 396–402, https://doi.org/10.1093/ijpp/riad019	Qualitative method	To explore perspectives and experiences of community pharmacists regarding addressing inappropriately prescribed analgesics	A qualitative study conducted with interviews of community pharmacists in England	Community pharmacists working in pharmacies in England	Pharmacists expressed mixed views on their involvement in tapering off inappropriately prescribed analgesics as part of their daily practice but mentioned that social and environmental barriers needed to be addressed to facilitate their involvement. Identified behavior change techniques provide evidence-based strategies to assist community pharmacists' involvement in identifying inappropriately prescribed analgesics.	Community pharmacists express the need to address social and environmental barriers to effectively participate in the management of inappropriately prescribed analgesics.
Owusu YB, Elkhalifa WH, Awaisu A, Kheir N. Assessment of Qatar community pharmacists' competence and practices related to renal and gastrointestinal adverse effects of nonprescription NSAIDs. Saudi Pharm J. 2022 Oct;30(10):1396-1404. doi: 10.1016/j.jsps.2022.06.011.	Qualitative method	To investigate community pharmacists' perceptions of opportunities and challenges in deprescribing NSAIDs in the context of public health	Qualitative study based on focus group discussions with community pharmacists	Community pharmacists in urban and rural areas	Pharmacists identified various opportunities for deprescribing NSAIDs, including patient education and collaboration with physicians, but also noted challenges such as lack of time and support. They highlighted the need for	Opportunities exist for community pharmacists to contribute to deprescribing NSAIDs, but significant barriers must be overcome, requiring training and systemic changes.

Article	Research Method	Purpose	Study Design	Target Group	Main Findings	Conclusion
					training and systemic changes to enhance their role in deprescribing.	
Clara H Heinrich, Maria D Donovan, Assessing community pharmacists' attitudes towards identifying opportunities for deprescribing in clinical practice in Ireland, International Journal of Pharmacy Practice, Volume 30, Issue 1, January 2022, Pages 28–35, https://doi.org/10.1093/ijpp/riab079	Systematic review	To explore the role of pharmacists in deprescribing and to identify barriers and facilitators.	Systematic review of quantitative and qualitative studies	Community and hospital pharmacists	Pharmacists can play a key role in deprescribing, but face barriers such as lack of confidence, inadequate communication with other healthcare providers, and insufficient training. Facilitators include professional development and support from healthcare teams.	Pharmacists have the potential to significantly contribute to deprescribing, but addressing barriers is essential for their effective involvement.
Gemmeke, M., Koster, E.S., Rodijk, E.A. et al. Community pharmacists' perceptions on providing fall prevention services: a mixed-methods study. Int J Clin Pharm 43, 1533–1545 (2021). https://doi.org/10.1007/s11096-021-01277-4	Qualitative method	To identify barriers and enablers to community pharmacists' involvement in deprescribing	Qualitative study using semi-structured interviews	Community pharmacists in the Netherlands	Barriers to deprescribing include lack of time, insufficient knowledge, and lack of collaboration with other healthcare providers. Enablers include professional training, supportive policies, and good communication with patients and healthcare teams.	Understanding and addressing the barriers and enablers is crucial for enhancing the role of community pharmacists in deprescribing.
Huffmyer MJ, Keck JW, Harrington NG, et al. Primary care clinician and community pharmacist	Mixed-methods study	To explore community pharmacists' perspectives on deprescribing	Mixed-methods study involving surveys and interviews	Community Pharmacists in Spain	Pharmacists identified both opportunities and challenges in deprescribing	Deprescribing in pain management presents both opportunities and

Article	Research Method	Purpose	Study Design	Target Group	Main Findings	Conclusion
perceptions of deprescribing. J Am Geriatr Soc. 2021; 69: 1686–1689. https://doi.org/10.1111/jgs.17092		in pain management			for pain management, such as the potential to improve patient outcomes and the difficulty in changing established prescribing practices. They suggested a need for better guidelines and training.	challenges for community pharmacists, with a need for improved guidelines and training.
Korenvain C, MacKeigan LD, Dainty KN, Guilcher SJT, McCarthy LM. Exploring deprescribing opportunities for community pharmacists using the Behaviour Change Wheel. Res Social Adm Pharm. 2020 Dec;16(12):1746-1753. doi: 10.1016/j.sapharm.2020.01.019.	Cross-sectional study	To assess the attitudes of community pharmacists towards deprescribing	Cross-sectional survey study	Community Pharmacists in Italy	The majority of pharmacists had positive attitudes towards deprescribing and recognized its importance, but reported barriers such as lack of time, patient resistance, and insufficient collaboration with other healthcare providers.	Positive attitudes towards deprescribing among community pharmacists indicate potential for involvement, but barriers need to be addressed.
