# Supplementary material for: Deprescribing NSAIDs: The Potential Role of Community Pharmacists
Source: Pharmacy (Basel). 2024 Jul 24;12(4):116. doi: 10.3390/pharmacy12040116 (PMC11358956; doi:10.3390/pharmacy12040116)
Supplement: Supplementary file 1 [file pharmacy-12-00116-s001.zip › Supplementary Material 2 .pdf]

Apotekfarmasøytens potensielle rolle i avmedisinering av NSAIDs

Ikke-steroide antiinflammatoriske midler (NSAIDs) er en gruppe medisiner som lindrer smerte og feber og reduserer betennelse. Eksempler på slike midler er ibuprofen, naproksen, og diklofenak. NSAIDs brukes til å behandle mild til moderat smerte som oppstår fra et bredt spekter av tilstander, inkludert hodepine, menstruasjonssmerter, migrene, artrose eller revmatoid artritt, forstuinger og belastningsskader, og tannpine. På grunn av deres utbredte bruk, er mange av disse medikamentene tilgjengelige på apotekene, både med og uten resept.

Til tross for deres effektivitet, representerer NSAIDs en betydelig risiko for pasienter på grunn av potensielle bivirkninger og interaksjoner, spesielt relatert til polyfarmasi og komorbiditet. Avmedisinering (engelsk: deprescribing), eller det å seponere eller redusere medisiner som ikke lenger anses som nødvendige eller som kan være skadelige, er en viktig prosess. Denne studien sikter mot å utforske og beskrive apotekfarmasøyters mulige roller i avmedisineringsprosessen av både reseptbelagte og reseptfrie NSAIDs i Norge. Dette er en sentral faktor i å håndtere polyfarmasi og forbedre pasientutfall ved smerteterapi. Din deltakelse vil gi verdifull innsikt i apotekfarmasøyters bidrag til pasientsikkerhet og effektivitet i legemiddelhåndteringen.

Vi håper at du kan ta deg tid til å svare på denne spørreundersøkelsen, som vil ta ca. 10 minutter. Resultatene vil bli brukt i mitt masterprosjekt i farmasi ved OsloMet, hvor jeg, Delsher Amedi, er masterstudent under veiledning av Parisa Gazerani. Takk for at du deltar.

Fraskrivelse:

Dette er en anonym nettbasert undersøkelse, og SIKT (kunnskapssektorens tjenesteleverandør, et statlig forvaltningsorgan under Kunnskapsdepartementet) er informert om denne anonyme undersøkelsen. Vi vil ikke samle inn noen av dine personlige eller sensitive opplysninger. For å delta i undersøkelsen må du være en farmasøyt med lisens eller autorisasjon til å arbeide i Norge, og du må være ansatt på et apotek, enten det er et kjedeapotek eller et privat apotek. Ved å delta i denne undersøkelsen gir du oss samtykke til å bruke svarene til akademiske formål, forskning og publisering av data i form av en vitenskapelig artikkel.

Har du lisens eller autorisasjon til å arbeide som farmasøyt i Norge?

Ja

Nei

Arbeider som farmasøyt på et apotek (kjedeapotek eller privat apotek)?

Ja

Nei

Takk for din interesse for å delta i undersøkelsen. Dessverre samler vi bare data fra apoteksfarmasøyter som jobber i kjede- eller private apotek.

SPØRRESKJEMA

Spørreskjemaet består av fem deler. Del 1 fokuserer på sosiodemografiske kjennetegn ved studiedeltakerne. Del 2 omhandler kunnskapen om avmedisinering. Del 3 dreier seg om selvtillit til å implementere avmedisinering av NSAIDs i praksis. Del 4 tar for seg holdninger knyttet til avmedisinering. Til slutt, del 5 utforsker utfordringer og muligheter for implementering av avmedisinering i praksis

Nettskjema

DEL 1: Sosiodemografiske kjennetegn ved studiedeltakerne

1 . Kjønn
Dette elementet vises kun dersom alternativet «Ja» er valgt i spørsmålet «Arbeider som farmasøyt på et apotek (kjedeapotek eller privat apotek)?»

Mann

Kvinne

Annet

Ønsker ikke å svare

2 . Alder
Dette elementet vises kun dersom alternativet «Ja» er valgt i spørsmålet «Arbeider som farmasøyt på et apotek (kjedeapotek eller privat apotek)?»

21-26 år

27-32 år

> 32 år

3 . Arbeidserfaring
Dette elementet vises kun dersom alternativet «Ja» er valgt i spørsmålet «Arbeider som farmasøyt på et apotek (kjedeapotek eller privat apotek)?»

Nyutdannet farmasøyt (0-1 år)

<5 år

5-10 år

>10 år

4 . Utdanningsnivå
Dette elementet vises kun dersom alternativet «Ja» er valgt i spørsmålet «Arbeider som farmasøyt på et apotek (kjedeapotek eller privat apotek)?»

Bachelor i farmasi

Master i farmasi

Annet

4.1 Vennligst beskriv nedenfor
Dette elementet vises kun dersom alternativet «Annet» er valgt i spørsmålet «4. Utdanningsnivå»

5 . Arbeidssted i Norge
Dette elementet vises kun dersom alternativet «Ja» er valgt i spørsmålet «Arbeider som farmasøyt på et apotek (kjedeapotek eller privat apotek)?»

Nord-Norge

Midt-Norge

Vestlandet

Østlandet

Sørlandet

6 . Farmasiutdannelsen oppnådd i Norge
Dette elementet vises kun dersom alternativet «Ja» er valgt i spørsmålet «Arbeider som farmasøyt på et apotek (kjedeapotek eller privat apotek)?»

Ja

Nei

I hvilket land fullførte du din farmasiutdanning fra?
Dette elementet vises kun dersom alternativet «Nei» er valgt i spørsmålet «6. Farmasiutdannelsen oppnådd i Norge»

DEL 2: Kunnskapen om avmedisinering

Vi vil nå stille noen kunnskapsspørsmål om avmedisinering

Hva er riktig om avmedisinering?

Beskriver en planmessig prosess for å avslutte eller redusere dosen av et legemiddel
Dette elementet vises kun dersom alternativet «Ja» er valgt i spørsmålet «Arbeider som farmasøyt på et apotek (kjedeapotek eller privat apotek)?»

Riktig

Galt

Vet ikke

Formålet er å redusere risikoen for alvorlige bivirkninger, bedre pasientens helse, og/eller redusere bruk av legemidler man ikke trenger
Dette elementet vises kun dersom alternativet «Ja» er valgt i spørsmålet «Arbeider som farmasøyt på et apotek (kjedeapotek eller privat apotek)?»

Riktig

Galt

Vet ikke

Bør kun initieres når en pasient opplever bivirkninger fra en medisin
Dette elementet vises kun dersom alternativet «Ja» er valgt i spørsmålet «Arbeider som farmasøyt på et apotek (kjedeapotek eller privat apotek)?»

Riktig

Galt

Vet ikke

En avgjørelse tatt av legen uten at pasient involveres
Dette elementet vises kun dersom alternativet «Ja» er valgt i spørsmålet «Arbeider som farmasøyt på et apotek (kjedeapotek eller privat apotek)?»

Riktig

Galt

Vet ikke

En prosess uavhengig av pasientens kliniske tilstand
Dette elementet vises kun dersom alternativet «Ja» er valgt i spørsmålet «Arbeider som farmasøyt på et apotek (kjedeapotek eller privat apotek)?»

Nettskjema

Riktig
Galt
Vet ikke

Avmedisinering er det samme som seponering

Dette elementet vises kun dersom alternativet «Ja» er valgt i spørsmålet «Arbeider som farmasøyt på et apotek (kjedeapotek eller privat apotek)?»

Riktig
Galt
Vet ikke

DEL 3: Selvtillit til å implementere avmedisinering av NSAIDs i praksis

I hvilken grad er du enig eller uenig i følgende påstander?

Jeg kan identifisere tilfeller hvor avmedisinering bør vurderes

Dette elementet vises kun dersom alternativet «Ja» er valgt i spørsmålet «Arbeider som farmasøyt på et apotek (kjedeapotek eller privat apotek)?»

Sterkt enig
Enig
Nøytral
Uenig
Sterkt uenig

Ved ekspedisjon av NSAIDs, hender det at jeg foreslår endringer i forhold til dosering og administrering

Dette elementet vises kun dersom alternativet «Ja» er valgt i spørsmålet «Arbeider som farmasøyt på et apotek (kjedeapotek eller privat apotek)?»

Sterkt enig
Enig
Nøytral
Uenig
Sterkt uenig

Jeg føler meg trygg på min kunnskap om bivirkninger og interaksjoner knyttet til NSAIDs når jeg veileder pasienter

Dette elementet vises kun dersom alternativet «Ja» er valgt i spørsmålet «Arbeider som farmasøyt på et apotek (kjedeapotek eller privat apotek)?»

Sterkt enig
Enig
Nøytral
Uenig
Sterkt uenig

Nettskjema

Jeg mener at min utdannelse innen farmasi har gitt meg nødvendige ferdigheter for å drøfte muligheter for avmedisinering med andre helsepersonell
Dette elementet vises kun dersom alternativet «Ja» er valgt i spørsmålet «Arbeider som farmasøyt på et apotek (kjedeapotek eller privat apotek)?»

Sterkt enig
Enig
Nøytral
Uenig
Sterkt uenig

Gjennom min utdanning i farmasi føler jeg meg godt forberedt på å diskutere avmedisinering med pasienter
Dette elementet vises kun dersom alternativet «Ja» er valgt i spørsmålet «Arbeider som farmasøyt på et apotek (kjedeapotek eller privat apotek)?»

Sterkt enig
Enig
Nøytral
Uenig
Sterkt uenig

DEL 4: Holdning knyttet til avmedisinering

I hvilken grad er du enig eller uenig i følgende påstander?

Å redusere legemiddelbruken vil bidra til å minske antallet av bivirkninger pasienter opplever som følge av deres medikamentterapi
Dette elementet vises kun dersom alternativet «Ja» er valgt i spørsmålet «Arbeider som farmasøyt på et apotek (kjedeapotek eller privat apotek)?»

Sterkt enig
Enig
Nøytral
Uenig
Sterkt uenig

Reduksjon i legemiddelbruk kan forbedre pasienters etterlevelse av medisinregimer
Dette elementet vises kun dersom alternativet «Ja» er valgt i spørsmålet «Arbeider som farmasøyt på et apotek (kjedeapotek eller privat apotek)?»

Sterkt enig
Enig
Nøytral
Uenig
Sterkt uenig

Det er mulig å identifisere muligheter for å redusere legemiddelbruk i mitt praksismiljø
Dette elementet vises kun dersom alternativet «Ja» er valgt i spørsmålet «Arbeider som farmasøyt på et apotek (kjedeapotek eller privat apotek)?»

Sterkt enig
Enig
Nøytral
Uenig
Sterkt uenig

Å tilegne meg ytterligere kunnskap om hvordan man innfører praksiser for avmedisinering på apotek står øverst på listen over mine umiddelbare opplæringsbehov

Dette elementet vises kun dersom alternativet «Ja» er valgt i spørsmålet «Arbeider som farmasøyt på et apotek (kjedeapotek eller privat apotek)?»

Sterkt enig
Enig
Nøytral
Uenig
Sterkt uenig

En del av farmasøytens rolle bør være å identifisere muligheter for avmedisinering under legemiddelgjennomgang

Dette elementet vises kun dersom alternativet «Ja» er valgt i spørsmålet «Arbeider som farmasøyt på et apotek (kjedeapotek eller privat apotek)?»

Sterkt enig
Enig
Nøytral
Uenig
Sterkt uenig

Hvis det er noen andre tanker, bekymringer eller erfaringer relatert til implementering av avmedisinering av NSAIDs som du ønsker å dele, vennligst skriv dem ned i boksen under.

Dette elementet vises kun dersom alternativet «Ja» er valgt i spørsmålet «Arbeider som farmasøyt på et apotek (kjedeapotek eller privat apotek)?»

DEL 5: Utfordringer og muligheter for implementering av avmedisinering i praksis, på en skala fra 1 til 5, der 5 er den største hindringen Tidsmangel – ikke nok tid til å vurdere resepter for muligheter til avmedisinering

Dette elementet vises kun dersom alternativet «Ja» er valgt i spørsmålet «Arbeider som farmasøyt på et apotek (kjedeapotek eller privat apotek)?»

Manglende økonomisk kompensasjon for gjennomgang av medikamentbruk

Dette elementet vises kun dersom alternativet «Ja» er valgt i spørsmålet «Arbeider som farmasøyt på et apotek (kjedeapotek eller privat apotek)?»

Apoteket mottar ikke ekstra betaling eller økonomisk insentiv for den tiden og innsatsen som kreves ved avmedisinering

Mangel på kunnskap om verktøy og metoder for avmedisinering

Dette elementet vises kun dersom alternativet «Ja» er valgt i spørsmålet «Arbeider som farmasøyt på et apotek (kjedeapotek eller privat apotek)?»

Nettskjema

Bekymringer knyttet til negative konsekvenser etter utførelse av avmedisinering

Dette elementet vises kun dersom alternativet «Ja» er valgt i spørsmålet «Arbeider som farmasøyt på et apotek (kjedeapotek eller privat apotek)?»

Kommunikasjon og tilgjengeligheten til forskrivere er problematisk

Dette elementet vises kun dersom alternativet «Ja» er valgt i spørsmålet «Arbeider som farmasøyt på et apotek (kjedeapotek eller privat apotek)?»

Forskriver er lite mottakelig for anbefalinger

Dette elementet vises kun dersom alternativet «Ja» er valgt i spørsmålet «Arbeider som farmasøyt på et apotek (kjedeapotek eller privat apotek)?»

Motvilje fra pasienten eller pårørende

Dette elementet vises kun dersom alternativet «Ja» er valgt i spørsmålet «Arbeider som farmasøyt på et apotek (kjedeapotek eller privat apotek)?»

Eksempel: manglende motivasjon, frykt for seponeringsreaksjoner

Hvis du mener det kan være andre utfordringer eller muligheter knyttet til avmedisinering, vennligst beskriv nedenfor

Dette elementet vises kun dersom alternativet «Ja» er valgt i spørsmålet «Arbeider som farmasøyt på et apotek (kjedeapotek eller privat apotek)?»

Nettskjema

Questionnaire (*Translated*)

The Potential Role of the Community Pharmacist in Deprescribing NSAIDs

Non-steroidal anti-inflammatory drugs (NSAIDs) are a group of medications that relieve pain and fever and reduce inflammation. Examples include ibuprofen, naproxen, and diclofenac. NSAIDs are used to treat mild to moderate pain resulting from a wide range of conditions, including headaches, menstrual pain, migraines, osteoarthritis, rheumatoid arthritis, sprains, strains, and dental pain. Due to their widespread use, many of these medications are available in pharmacies, both with and without prescription.

Despite their effectiveness, NSAIDs pose a significant risk to patients due to potential side effects and interactions, especially related to polypharmacy and comorbidity. Deprescribing, or discontinuing or reducing medications that are no longer considered necessary or that may be harmful, is an important process. This study aims to explore and describe community pharmacists' potential roles in the deprescribing process of both prescription and over-the-counter NSAIDs in Norway. This is a crucial factor in managing polypharmacy and improving patient outcomes in pain therapy. Your participation will provide valuable insights into community pharmacists' contributions to patient safety and the efficiency of medication management.

We hope you can take the time to respond to this survey, which will take about 10 minutes. The results will be used in my master's project in pharmacy at OsloMet, where I, Delsher Amedi, am a master's student under the supervision of Parisa Gazerani. Thank you for participating.

Disclaimer:

This is an anonymous online survey, and SIKT (the knowledge sector service provider, a government agency under the Ministry of Education and Research) has been informed of this anonymous survey. We will not collect any of your personal or sensitive information. To participate in the survey, you must be a licensed pharmacist authorized to work in Norway, and you must be employed at a pharmacy, either a chain pharmacy or a private pharmacy. By participating in this survey, you consent to the use of your responses for academic purposes, research, and publication of data in the form of a scientific article.

Do you have a license or authorization to work as a pharmacist in Norway?

- Yes
- No

Are you working as a pharmacist at a pharmacy (chain pharmacy or private pharmacy)?

- Yes
- No

Thank you for your interest in participating in the survey. Unfortunately, we only collect data from community pharmacists working in chain or private pharmacies.

Questionnaire (Translated in English)

The questionnaire consists of five parts. Part 1 focuses on sociodemographic characteristics of the study participants. Part 2 concerns knowledge of deprescribing. Part 3 deals with confidence in implementing deprescribing of NSAIDs in practice. Part 4 addresses attitudes towards deprescribing. Finally, part 5 explores challenges and opportunities for implementing deprescribing in practice.

PART 1: Sociodemographic Characteristics of the Study Participants

1. **Gender**
    - Male
    - Female
    - Other
    - Prefer not to answer
2. **Age**
    - 21-26 years
    - 27-32 years
        - 32 years
3. **Work Experience**
    - Newly graduated pharmacist (0-1 year)
    - < 5 years
    - 5-10 years
        - 10 years
4. **Educational Level**
    - Bachelor in Pharmacy
    - Master in Pharmacy
    - Other 4.1 **Please specify below**
5. **Workplace in Norway**
    - Northern Norway
    - Central Norway
    - Western Norway
    - Eastern Norway
    - Southern Norway
6. **Pharmacy Education Completed in Norway**
    - Yes
    - No 6.1 **In which country did you complete your pharmacy education?**

PART 2: Knowledge of Deprescribing

We will now ask some knowledge questions about deprescribing.

1. **What is correct about deprescribing?**
    - Describes a systematic process to discontinue or reduce the dose of a medication
        - Correct
        - Incorrect

  - Don't know

2. **The purpose is to reduce the risk of serious side effects, improve the patient's health, and/or reduce the use of unnecessary medications.**
   - Correct
   - Incorrect
   - Don't know
3. **Should only be initiated when a patient experiences side effects from a medication.**
   - Correct
   - Incorrect
   - Don't know
4. **A decision made by the doctor without involving the patient.**
   - Correct
   - Incorrect
   - Don't know
5. **A process independent of the patient's clinical condition.**
   - Correct
   - Incorrect
   - Don't know
6. **Deprescribing is the same as discontinuation.**
   - Correct
   - Incorrect
   - Don't know

PART 3: Confidence in Implementing Deprescribing of NSAIDs in Practice

To what extent do you agree or disagree with the following statements?

1. **I can identify cases where deprescribing should be considered.**
   - Strongly agree
   - Agree
   - Neutral
   - Disagree
   - Strongly disagree
2. **When dispensing NSAIDs, I sometimes suggest changes regarding dosage and administration.**
   - Strongly agree
   - Agree
   - Neutral
   - Disagree
   - Strongly disagree
3. **I feel confident in my knowledge of the side effects and interactions related to NSAIDs when advising patients.**
   - Strongly agree
   - Agree
   - Neutral
   - Disagree
   - Strongly disagree

4. **I believe my education in pharmacy has provided me with the necessary skills to discuss deprescribing with other healthcare professionals.**
    - o Strongly agree
    - o Agree
    - o Neutral
    - o Disagree
    - o Strongly disagree
5. **Through my pharmacy education, I feel well-prepared to discuss deprescribing with patients.**
    - o Strongly agree
    - o Agree
    - o Neutral
    - o Disagree
    - o Strongly disagree

PART 4: Attitudes Towards Deprescribing

To what extent do you agree or disagree with the following statements?

1. **Reducing medication use will help to reduce the number of side effects patients experience as a result of their medication therapy.**
    - o Strongly agree
    - o Agree
    - o Neutral
    - o Disagree
    - o Strongly disagree
2. **Reducing medication use can improve patients' adherence to medication regimens.**
    - o Strongly agree
    - o Agree
    - o Neutral
    - o Disagree
    - o Strongly disagree
3. **It is possible to identify opportunities to reduce medication use in my practice environment.**
    - o Strongly agree
    - o Agree
    - o Neutral
    - o Disagree
    - o Strongly disagree
4. **Acquiring additional knowledge on how to implement deprescribing practices in the pharmacy is at the top of my immediate training needs.**
    - o Strongly agree
    - o Agree
    - o Neutral
    - o Disagree
    - o Strongly disagree
5. **Part of the pharmacist's role should be to identify opportunities for deprescribing during medication reviews.**
    - o Strongly agree

- Agree
- Neutral
- Disagree
- Strongly disagree

6. **If you have any other thoughts, concerns, or experiences related to the implementation of deprescribing of NSAIDs that you would like to share, please write them in the box below.**

PART 5: Challenges and Opportunities for Implementing Deprescribing in Practice

On a scale of 1 to 5, where 5 is the greatest barrier:

1. **Lack of time - not enough time to review prescriptions for deprescribing opportunities.**
2. **Lack of financial compensation for medication reviews.**
   - The pharmacy does not receive extra payment or financial incentive for the time and effort required for deprescribing.
3. **Lack of knowledge about tools and methods for deprescribing.**
4. **Concerns about negative consequences after deprescribing.**
5. **Communication and availability of prescribers is problematic.**
6. **Prescriber is unreceptive to recommendations.**
7. **Resistance from the patient or relatives.**
   - Example: lack of motivation, fear of withdrawal reactions
8. **If you believe there are other challenges or opportunities related to deprescribing, please describe them below.**
